# Management of Leptomeningeal Metastases in Non-oncogene Addicted Non-small Cell Lung Cancer

**DOI:** 10.3389/fonc.2018.00278

**Published:** 2018-07-27

**Authors:** Ana Turkaj, Anna M. Morelli, Tiziana Vavalà, Silvia Novello

**Affiliations:** ^1^Department of Oncology, University of Torino, Ospedale San Luigi Gonzaga, Orbassano, Italy; ^2^SC of Oncology, ASL CN1, Ospedale Civile di Saluzzo, Saluzzo, Italy

**Keywords:** non-small cell lung cancer (NSCLC), brain metastases, leptomeningeal metastases, chemotherapy, intra-thecal chemotherapy, immunotherapy, radiotherapy

## Abstract

Brain metastases in non-small cell lung cancer (NSCLC) patients are more often detected due to imaging modalities improvements but also emerge because of improved treatments of the primary tumor which lead to a longer survival. In this context, development of leptomeningeal metastases (LM) is a devastating complication and its prognosis remains poor despite advances in systemic and local approaches. Histology characterization of NSCLC and molecular expression influence LM management. For those with “oncogene addiction,” new generation epidermal growth factor receptor (EGFR) and anaplastic lymphoma kinase (ALK) tyrosine kinase inhibitors (TKIs) were developed to strongly penetrate the blood-brain barrier (BBB) with the aim to prevent central nervous system cancer dissemination, eventually impacting on LM appearance and its subsequent management. Systemic chemotherapy, often combined with intrathecal chemotherapy (when possible), was one of common indications for lung cancer patients affected by LM, without driver mutations and a good performance status but currently, with the advent of innovative systemic approaches treatment solutions in this subgroup of patients are rapidly evolving. Whole brain radiation therapy (WBRT) is the conventional treatment for patients with brain metastases. Furthermore, modern radiation techniques, as stereotactic radiotherapy (SRT), improve outcomes in those cases with a limited number of lesions. However, LM represent a minority of CNS metastases and few literature data are available to drive the radiotherapy approach. Considering all relevant progress made in this setting, after a literature review, the aim of this paper is to discuss about recent developments and therapeutic options in LM management of non-oncogene addicted NSCLC.

## Introduction

Non-small cell lung cancer (NSCLC) is characterized by a high incidence of central nervous system (CNS) dissemination, with approximately 40% of patients developing brain metastases (BM) in the course of their disease (1, 2) and leptomeningeal metastases (LM) appearance in a smaller percentage (3–5%) (3). Particularly, LM incidence among NSCLC patients is 3.8% more frequent in adenocarcinoma subtype, with about a third of those patients having concomitant BM (4). LM usually manifest as a late complication, which have been reported to emerge as late as up to 112 months after initial diagnosis (5). LM cases are increasing in incidence as a result of improved survival in subgroups of patients with targetable mutations treated with molecular therapies, but also because of modern neuro-imaging tools, able to clearly identify even small foci of meningeal dissemination (6–8). Median survival of NSCLC patients affected by LM is particularly poor even with signs of improvement, from a historical median survival of 1–3 to 3–11 months with novel therapies and integration of local and systemic treatments (9). Specific treatments of LM depends on histology characterization of NSCLC, molecular expression, time of appearance and patient's performance status. Molecularly targeted therapies and immunotherapy showed antitumor activity for brain metastases although effectiveness in cytologically confirmed and symptomatic LM is still limited or unknown (3). Systemic and intrathecal (IT) chemotherapy with site-specific radiotherapy are usually applied, particularly in non-oncogene addicted NSCLC, while up to one-third of the patients are treated with best supportive care alone. Despite the lack of the standard treatments, active treatments have been associated independently with longer overall survival (OS) (10). Recent advances in the understanding of LM biology in NSCLC patients along with the development of highly active targeted drugs for tumors with specific genetic alterations, helped to redefine the prognosis in this subgroup of patients and the same evolution is largely awaited in those NSCLC patients without oncogene addiction (5, 11–14). Based on literature review, this paper aims to discuss about recent developments and therapeutic options of LM in NSCLC patients without driver mutations.

## Biology of blood brain barrier and drug delivery

The blood–brain barrier (BBB) is constituted by a continuous stratum of endothelial cells connected by tight junctions surrounded by pericytes and perivascular end feet of astrocytes, thus being a highly selective barrier which separates systemic circulation from cerebrospinal fluid (CSF) (15).

BBB maintains CNS homeostasis by enabling the transport of selected substances only, through a combined result of influx and efflux mechanisms. Therapeutic efficacy in this area is determined by whether drug concentrations can be achieved in the CSF and these differ as a result of multiple conditions. The ability of a drug to cross the BBB is substantially improved by particular physic-chemical properties, including low potential for active efflux, few rotatable bonds, small polar surface area, few hydrogen bond donors (9). For instance, anticancer therapeutics (tyrosine kinase inhibitors or chemotherapeutic agents), are substrates of efflux transport proteins, such as P-glycoprotein, which is responsible for the transport of most drugs outside the intracranial region. Most chemotherapy agents have low CSF concentrations, with relevant liquor permeability reported only for temozolomide, methotrexate and topotecan (16–19), however predicted CNS penetration does not necessary correlate with known response rates to chemotherapeutic agents. Moreover, free diffusion of molecules across the BBB requires both lipophilicity and low molecular weight (less than 0.5 kDa): chemotherapy drugs are usually larger than 150 kDa, hydrophilic and frequently protein-bound molecules, therefore unable to penetrate an intact BBB (20–23). In this context, growing scientific evidence highlights that CNS metastases cause BBB interruption; this process, probably due to tumor neo-angiogenesis, lead to generate new vessels lacking of structural and physiological features of normal BBB, thus favoring the passage of drugs into the brain (24, 25). The same hypothesis emerges after whole brain radiation (WBRT) approach, thus providing a biologic rationale for using concomitant or sequential systemic and local treatments in these cases (26, 27).

## Diagnosis of leptomeningeal metastases

LM involve penetration of inner layers of meninges and subarachnoid space in which CSF circulates. Its diagnosis is specifically based on three different assessments: clinical signs and symptoms, CSF cytological examinations and neuro-radiological imaging.

Early clinical presentation can be subtle and may include headaches and back pain, cranial nerve deficits, cauda equine symptoms or signs, visual disturbances, diplopia, hearing loss and neurocognitive syndromes. In later stages, symptoms related to elevated intracranial pressure could occur (28, 29). Cytological identification of malignant cells in CSF is the gold standard for diagnosis of LM. The sensitivity of the initial lumbar puncture was reported to be as low as 50%, with a potential increase to 75–85% with a second CSF analysis (30). A meningeal biopsy is rarely needed to confirm a clinical suspect. A recent study performed by Jiang et al. demonstrated that the use of next-generation sequencing (NGS) performed on cerebrospinal fluid circulating tumor cells (CSF-CTC) may be a more sensitive and an effective way to diagnose LM, serving also as a liquid biopsy for gene profiles in NSCLC patients with LM (31). Besides clinical and cytological diagnosis, brain and spine imaging are able to identify involved sites, even in cytology-negative cases (32).

Magnetic resonance imaging (MRI), ideally with a 3 Tesla scanner is the most useful imaging modality for the detection of LM. Both T1 with and without contrast enhancement and high resolution T2 fluid attenuated inversion recovery sequences post-contrast are important in establishing a radiological diagnosis of LM.

Particularly the disruption of BBB in presence of CNS metastases is often evidenced by peritumoral edema and accumulation of contrast during the imaging scans and, as more recently observed, penetration of CNS metastases is identified by nuclear medicine tracers, such as 18-Sodium Fluoride (33).

The EANO-ESMO clinical practice guidelines propose to classify LM by using two major criteria, being “type I” those LM when the diagnosis has been verified citologically or histologically and “type II” in the absence of verification. While on the basis of the neuroimaging findings: linear leptomeningeal disease (type A), nodular leptomeningeal disease (type B), both (type C) or neither nor, e.g., no neuroimaging evidence of LM except possibly hydrocephalus (type D) (34).

## Treatment options of leptomeningeal metastases

Treatment of LM is preferentially multidisciplinary and mostly indicated when diagnosis is unequivocal or symptoms are strongly suggestive, in case of negative cytology.

The goals of treatment in patients with LM are to improve or stabilize the neurologic status of the patient, maintain or regain quality of life and optimally to prolong survival together with marginal toxicity. Limited data are available to establish treatment recommendations in the management of LM: no randomized trials proved a survival benefit of a specific treatment modality and, accordingly, the optimal strategy is still poorly defined, particularly in non-oncogene addicted NSCLC. In this last setting of patients, palliative radiotherapy to symptomatic sites of disease, cytotoxic chemotherapies, intrathecal therapy (or a combination of these modalities) are traditionally considered, with the new innovative immunotherapy chance (35–39).

### Radiation therapy

In the era of improved radiation modalities, local treatment of BM is rapidly improving: a growing amount of data support an integrate use of WBRT or stereotactic radiosurgery (SRS) with systemic treatment in a variety of clinical scenarios, together with alternative radiation approaches, such as intensity modulated radiation therapy (IMRT) or even proton beam therapy, when applicable (40–42).

LM represent a minority of CNS metastases (11–20%) (43, 44) and less data are available to inform decisions about therapy. RT is mainly administered for symptoms alleviations, CSF flow correction or for debulking to facilitate chemotherapy.

WBRT is typically used in cases of concurrent brain metastases or major meningeal cerebral involvement (45). For BM dose and fractionation scheme is at the discretion of the treating radiation oncologist, though most commonly used dose and fractionation schemes are 20 Gy in 5 fractions of 4 Gy (standard schedule in Europe) and 30 Gy in 10 fractions of 3 Gy (46). For LM focal radiation therapy is recommended on symptomatic, bulky or obstructive sites and the dose depends on performance status (20–40 Gy in 5–20 fractions), volume to treat and available techniques (47). Different studies reported a survival difference in favor of patients with better performance status after various treatments, including WBRT (48, 49). Gani et al. evidenced that WBRT alone in patients with LM from breast and lung cancer is an effective palliative treatment for patients unfit for chemotherapy and poor performance status (49). As tumor dissemination affects the whole CSF compartment, according to some studies, the complete craniospinal axis should be encountered as target volume. Favorable results have been reported in small series. To determine the effects of craniospinal irradiation (CSI) a retrospective study of 16 patients with LM (mostly from breast and lung cancer) was conducted by Hermann et al. In this study, ten patients were additionally treated with intrathecal methotrexate. The authors conclude that craniospinal radiotherapy is feasible and effective for palliative treatment of LM (36).

However, craniospinal irradiation (CSI) is generally not recommended, as it is assumed to cause substantial myelotoxicity. In fact, in his review, Chamberlain stated that “whole neuroaxis radiation of the craniospinal axis is rarely indicated in the treatment of LM in solid tumors” (50).

Conformal radiotherapy may help to limit bone marrow and neurotoxicity making focal radiotherapy better tolerated. Proton therapy is only available in a few centers, but this approach promises further reduction of toxicity and effectiveness from CSI (51, 52).

Focal RT administration in fractionated regimens, such as involved-field or stereotactic RT or administered in single fractions (radiosurgery), can be used to treat nodular disease and symptomatic cerebral or spinal sites (34). By contrast, stereotactic radiosurgery (SRS), which is a radiation therapy technique in which multiple focused radiation beams intersect over a target, results in delivery of a highly conformal, high-dose of radiation to the target and minimal radiation to the surrounding normal tissues. In patients with BM a Gamma Knife Radiosurgery (GKRS) is typically used, depending upon the volume and location prescription doses typically range from 15 to 24 Gy for single fraction session (53). GKRS allow to achieve high rates of local control, and is able to delay the need for WBRT thus avoid potential neurocognitive toxicities, although a phase 2 RTOG study suggested that concomitant administration of memantine together with WBRT may reduce and delay subsequent cognitive consequences (54). Few studies have reported on the role of SRS in the setting of LM (55, 56). In the small and heterogeneous study by Wolf et al. the prescription tumor margin dose was a median of 16 Gy (11–20 Gy) to the 50–80% isodose volumes. The authors suggested SRS capable to provide high rates of local control for restricted LM with a median survival of 10 months and with 60% of the population alive at 6 months. SRS for focal LM is preferable in those patients who are eligible for systemic therapy, including immuno-therapies and targeted therapies, which can potentially further prolong overall survival.

Involved field radiotherapy (IFRT) is considered to be the standard of care for palliative treatment of LM. Relevant to this, the US National Comprehensive Cancer Network (NCCN) 2017 guidelines for management of LM recommend Intrathecal Chemotherapy (IT) in combination with IFRT in patients with good prognosis disease (as defined by high performance status, non-fixed neurologic deficits, minimal systemic disease, and reasonable options for systemic disease treatment). Patients not meeting criteria for good prognosis are recommended to undergo IFRT to symptomatic sites or best supportive care (45). Up to now, no randomized clinical trial to assess the efficacy and tolerance of RT in LM have been conducted, however use of radiation therapy in NSCLC patients with LM, particularly in those not presenting driver mutations, needs to be better defined in clinical trials. Concomitant strategies with ITC are currently not considered as standard care due to the toxicity profile. Phase II clinical trial of combination therapy with involved field RT combined with concurrent intrathecal-MTX or intrathecal-ARA-C is currently underway (57).

Table 1 summarize the published studies from 2000: different types of radiation modality on different histologies in patients with LM are included (36, 49, 56, 58–61).

**Table 1:** Selected trials published of radiation modality treatment with or without chemotherapy/immunotherapy association in patients with LM/BM [BM, brain metastasis; LM, leptomeningeal metastasis, Involved-field radiotherapy (IF-RT), Intrathecal chemotherapy (IC), Craniospinal Irradiation (CSI) Methotrexate (MTX), Non available (NA), Not reached (NR), Patients (pt)].

**Author and year**	**Number of patients**	**Histology**	**CNS metastasis**	**Status of EGFR/ALK NSCLC**	**Treatment**	**Median overall survival (mOS) (weeks)**
Wolf et al. (56) (retrospective)	16	8 NSCLC 5 breast cancer 3 other	LM	4 EGFR mutant 1 ALK-rearranged 3 no mutation	SRS (5 pt had prior WBRT)	40
Pan et al. (58) (prospective phase 2 study)	59	32 NSCLC 10 SCLC 11 breast cancer 6 Other	LM	NA	Concomitant IF-RT + IC MTX	26
Ozdemir et al. (59) (retrospective)	51	30 SCC 21 Adenocarcinoma	LM	NA	WBRT	15, 6
Brower et al. (60) (retrospective)	124	32 NSCLC 22 breast cancer 21 SCLC 49 other	LM	NA	Chemotherapy + WBRT	9, 2
Gani et al. (49) (retrospective)	27	20 breast cancer 7 lung cancer	LM	NA	WBRT	8, 1
Hermann et al. (36) (retrospective)	16	9 breast cancer 5 lung cancer 2 other	LM	NA	CSI (10 pt CSI + ITC MTX)	12 8 RT alone 16 RT-ITC
Goldberg et al. (61) (two-cohort phase II trial)	36	18 melanoma 18 NSCLC	BM	KRAS mutant 4 EGFR mutant 1 ALK-rearranged 1 PD-L1 positive 18	Pembrolizumab 20 pt prior CNS therapy (9 lesions WBRT 7 lesions SRS)	Melanoma (NR) NSCLC 30, 8

In the last few years, understanding of immune system's role in the response to ionizing radiation is progressively raising, novel opportunities to study how to combine immunotherapy with radiation-induced cell killing are revolutionizing cancer treatment. Accumulating preclinical and clinical data showed that combination of radiation techniques with immunotherapy stimulates immune response, improves locoregional and distant control finally resulting in better OS. Radiation appears to stimulate the immune system through multiple mechanisms, including the increase of the tumor-associated antigens (TAAs) availability, improving antigen presentation and subsequent stimulation of effector T cells, and enhancing infiltration of dentritic cells and T cells into the tumor microenvironment. The limited evidence for immunotherapy to date in the treatment of BM and LM stems from the deliberate exclusion of patients with active brain metastases from many large randomized trial assessing drug efficacy (61–63). However, comparable efficacy of immunotherapy agents in the brain and at extracerebral sites with radiation therapy has been recently reported by an ongoing study from Goldberg et al. In this initial analyses 36 cases were considered, 18 with melanoma and 18 with NSCLC. Patients were treated with pembrolizumab 10 mg/kg every 2 weeks until progression, no target lesions were previously resected, 20 patients received some form of local CNS therapy prior to enrollment (9 and 7 lesions had been treated respectively with WBRT and SRS, 75 lesions were untreated). The primary endpoint was BM response rate and the initial results presented demonstrated a systemic benefit from immunotherapy in patients with metastatic melanoma and NSCLC (61). Combining immunotherapeutic agents with stereotactic radiosurgery appears to enhance both local and distant control, and result in better survival (42).

Finally, other types of RT in patients without obstruction to CSF flow, as radioimmunotherapy (RIT) consisting of intra CSF administration of radioisotopes like iodine-131 (^131^I) and yttrium-90 (^90^Y) with radiolabeled antibodies as HMFG1, 3F8 and 8H9 have been utilized, but further improvement in the pharmacokinetic modeling of CNS RIT modality should refine this emerging therapy to fit the clinical context (63, 64).

### Systemic therapy

In the last decade, the advent of EGFR- tyrosine kinase inhibitors (TKIs) has improved the prognosis of NSCLC patients harboring EGFR mutations on both CNS metastases and extracranial disease, by contrast non-oncogene addicted NSCLC prognosis remains extremely poor.

The role of chemotherapy for patients with CNS metastases from NSCLC has been neglected for years, because of prevailing belief that chemotherapeutic drugs cannot cross at all the BBB. A Platinum based-combination (preceded or not by a local radiation treatment) is the mainstay of treatment in advanced non-small cell lung cancer (aNSCLC) with BM and or LM NSCLC at diagnosis without oncogenic driver mutations or *programmed death*-*ligand 1 (PD-L1)* tumor proportion score (TPS) values ≥ 50% (65–73). Table 2 summarize platinum-based chemotherapy for NSCLC patients with CNS (65–73).

**Table 2:** Platinum-based chemotherapy trials for CNS from NSCLC (BM, brain metastasis; LM, leptomeningeal metastasis).

**Author and year**	**Number of patients**	**CNS metastasis**	**Histology**	**Treatment**	**Intracranial RR (IRR, %)**	**Median overall survival (mOS) (weeks)**
Robinet et al. (71)	171	BM	NSCLC	Cisplatin-Vinorelbine	33	24
Franciosi et al. (68)	43	BM + LM	NSCLC	Cisplatin-Etoposide	37	32
Barlesi et al. (72)	43	BM	NSCLC	Cisplatin-Pemetrexed	41.9	29.6
Cotto et al. (69)	31	BM	NSCLC	Cisplatin-Fotemustine	23	16
Fujita et al. (67)	30	BM	NSCLC	Cisplatin-Ifosfamide-Irinotecan	50	56
Bailon et al. (73)	26	BM	NSCLC	Carboplatin-Pemetrexed	40	39
Cortes et al. (65)	26	BM	NSCLC	Paclitaxel Cisplatin/Vinorelbine-Gemcitabine	38	21.4
Minotti et al. (66)	23	BM	NSCLC	Cisplatin-Teniposide	35	21
Bernardo et al. (70)	22	BM	NSCLC	Carboplatin-Vinorelbine-Gemcitabine	45	33

Pemetrexed is a compound currently approved both in combination with platinum in first-line setting and as a single agent in maintenance or second line setting for the treatment of non-squamous cell carcinoma (74–77). Although a penetration of CNS of less 5%, pemetrexed demonstrated a consistent activity against BM from NSCLC with an intracranial RR of 30.8−41.9% and an overall clinical benefit of 63% without specific data for LM (72, 73, 78, 79) Bevacizumab is an anti-VEGF monoclonal antibody approved in patients affected by locally advanced or metastatic non-squamous cell carcinoma (80). Recent reports showed that bevacizumab improve intra-tumor penetration of other chemotherapeutic agents, such as carboplatin or paclitaxel by normalizing angiogenesis at the tumor site with a low incidence of CNS hemorrhage (81, 82). Additionally, bevacizumab appeared to alter neuroimaging characteristics of LM, confounded diagnosis and possibly also influenced the pattern of tumor spread of LM (83). Bevacizumab is also known as a “steroid-sparing” drug, that allows reduction in steroid dosage and achieves reductions in mass effect and peritumoral edema getting better control of neurological symptoms (84). In those patients with squamous histology and low PD-L1 expression, gemcitabine and taxanes are largely prescribed, also in patient with BM, but no specific data about LM are available (70, 85, 86).

For both squamous and non-squamous cell carcinoma without oncogene-addiction and TPS ≥ 50%, pembrolizumab represents the standard of care in the first-line setting. Data on the efficacy of immunotherapy for BM or LM are currently limited, because of the exclusion of these patients from clinical trials (38, 87–89). In the trial by Goldberg et al., already mentioned above, 36 patients with BM were treated with pembrolizumab. Among patients with NSCLC, BM response rate was 33% and treatment-related serious adverse events were rare. Several aspects of study population need to be considered looking at the results: patients eligible for this trial were those with BM < 20 mm, asymptomatic and not requiring corticosteroids to control neurologic symptoms, without autoimmune disease and with no prior treatment with agents targeting PD-1/PD-L1 (61). Only preliminary data are available on efficacy of anti-programmed cell death 1 (PD-1) agents (nivolumab, pembrolizumab) or anti-PD ligand 1 (atezolizumab) in NSCLC patients with BM. A review on efficacy and safety of nivolumab conducted by Dudnik in five patients with aNSCLC with new/progressing asymptomatic CNS metastasis (including patient with LM) suggested that immune-check inhibitors might have an intracranial activity. Two intracranial responses were observed, while stabilization of LM was achieved in 10 weeks (90). In the Italian Nivolumab Squamous NSCLC Expanded Access Program, 38 patients with treated and asymptomatic BM were included, none of them with LM. In this subgroup of patients immunotherapic agent obtained a disease control rate equal to 47.3% (91). On the other hand, Otsubo et al., reported a case report of the development of LM during a pronounced response of the primary tumor to pembrolizumab therapy in a NSCLC patient (92). Relevant to this, in the era of molecular oncology it will be important to consider genomic differences in systemic malignancies that can implicate a distinct immune response.

Looking at other type of compounds, a phase II trial with abemaciclib (orally bioavailable inhibitor of cyclin-dependent kinases 4 and 6) (93) is ongoing in patients with LM from NSCLC and solid tumors (94).

### Intra-thecal chemotherapy

Intrathecal administration is the most common method to deliver chemotherapeutic agents in non-nodular and non-bulky LM in solid tumors, although efficacy compared to systemic administration and choice of regimen are poorly understood due to limited randomized controlled trials (95). Systemic chemotherapy, which may be combined with intrathecal chemotherapy, remains standard treatment for lung cancer patients with LM and a good-risk profile (45).

Several retrospective studies demonstrated a survival benefit from IT-therapy. Because of the paucity of available patients, LM studies often accrue multiple primary histologies. Most reports of intrathecal LM treatments include patients who simultaneously receive systemic agents (29, 95–98). Although the compounds routinely used for intra-CSF treatment do not have a key role as single agents for systemic treatment of most common cancers causing LM, three agents are commonly prescribed for the intrathecal treatment of LM: methotrexate (98), cytarabine (including liposomal cytarabine) (99, 100) or thioTEPA (8). Several schedules have been proposed, without agreement on optimal dose, frequency of administration or optimal duration of treatment. No intra-CSF agent has shown a significant survival advantage over another (8, 99, 100). Up to now most of the patients are treated until progression or for 1 year, if tolerated. In the absence of evidence from appropriate clinical trials, clinical symptoms, MRI and CSF findings, as well as tolerance of treatment, guide individual decisions on the duration of treatment (34). Pemetrexed is a newer generation multi-targeted anti-folate agent and, compared with methotrexate, has better tolerability, exhibits a more favorable side effect profile, and possesses fewer-drug interactions (78). A phase I trial (NCT03101579) is ongoing to define safety profile and clinical response rate associated with this specific intrathecal therapy (101).

## Conclusion

LM is undoubtfully a serious complication in NSCLC patients. Prognosis remains poor, even with the use of personalized treatments, principally due to low penetration into the CSF of currently used agents. To our knowledge no randomized trial has demonstrated a clear survival benefit of any single modality in the treatment of LM. The optimal treatment strategy involves a multi- disciplinary approach. The increasing prevalence of LM warrants further investigation into therapies and prognostic variables to serve as a guide for treatment recommendations and patients counseling. An improved understanding of the biologic mechanisms underlying tumor CNS metastases and novel available diagnostic tools will allow for patient-tailored treatment strategies. Future well-designed randomized controlled studies are needed to evaluate the efficacy of chemotherapeutics, immunotherapies and radiation treatment in this specific subgroup of patients and, more specifically, in those without an “oncogene addiction.”

## Author contributions

AT and AM wrote the manuscript with support from TV. SN supervised the manuscript.

### Conflict of interest statement

The authors declare that the research was conducted in the absence of any commercial or financial relationships that could be construed as a potential conflict of interest.
